# Engineering a Hyper-Adherent *E. coli* Nissle Probiotic Strain That Reduces Intestinal Carriage of *Salmonella* Pathogens in Poultry

**DOI:** 10.3390/foods15203626

**Published:** 2026-10-12

**Authors:** M. Aaron Baxter, Kelsey Greenwood, Kristi L. Anderson, Steve A. Carlson, Bradley D. Jones

**Affiliations:** 1 Department of Biomedical Sciences, Grand Valley State University, Allendale, MI 49401, USA; baxteraa@gvsu.edu; 2 Department of Microbiology and Immunology, University of Iowa Carver College of Medicine, Iowa City, IA 52242, USA; kelsey.greenwood@louisville.edu; 3 Department of Biomedical Sciences, Iowa State University College of Veterinary Medicine, Ames, IA 50011, USA

**Keywords:** probiotic, Salmonella, poultry, microbiome, adherence, competitive exclusion

## Abstract

Poultry meat and eggs are major sources of *Salmonella* foodborne illness, due to carriage of these bacterial pathogens in the intestinal microbiome of chickens, which is an ongoing food safety issue throughout the world. Reducing or eliminating the carriage of *Salmonella* in poultry would significantly improve food safety and reduce cases of human salmonellosis. This study represents a new approach to reduce or eliminate carriage of *Salmonella* in poultry flocks. We have created an *E. coli* Nissle 1917 probiotic strain that expresses a *Salmonella* factor with high levels of adherence. The *E. coli* Nissle 1917 scaffold has been used for many years as a probiotic to treat human intestinal disorders and is designated GRAS by the FDA. Our probiotic strain displays high levels of adherence to host cells, in fact, higher levels of adherence than a *Salmonella* strain carrying the same type 1 fimbrial genes. We demonstrate that the probiotic strain significantly outcompetes pathogenic *Salmonella* strains for adherence to tissue culture cells (more than 100-fold; >2 logs), and in vivo experimental challenges revealed that the probiotic strain mediates a significant exclusion (more than a 10-fold reduction; >1 log) of *Salmonella* from the intestines of broilers, layers, and turkeys.

## 1. Introduction

Pathogenic *Salmonella* species are an important cause of foodborne illness in the U.S. with an estimated 1.35 million human infections and 26,500 hospitalizations each year, with an associated cost of USD 4.1 billion annually in productivity (https://www.usda.gov/media/press-releases/2022/10/14/usda-releases-proposed-regulatory-framework-reduce-salmonella, accessed on 25 June 2026). Approximately 30% of human cases of *Salmonella* gastroenteritis are linked to poultry consumption, making the reduction of CFUs of pathogenic *Salmonella* carried in poultry an urgent food safety priority. Achievement of this food safety goal will substantially reduce human salmonellosis infections and save the associated costs in health and financial expenses. Efforts to identify and characterize the factors of *Salmonella* that allow this pathogen to cause human disease are extensive. Research has identified and characterized genes necessary for cell invasion (SPI-1 genes and associated effectors) [1,2,3,4], survival within host cells, such as macrophages (SPI-2 genes and associated effectors) [5,6,7], and survival within the human host that are encoded on additional pathogenicity islands [6,8,9] as well as the virulence plasmid [8]. Many other genes have also been studied that encode LPS, serum resistance that allows the bacteria to survive and persist in a host [4,8]. While these studies yielded enormous information on the biology and virulence strategy of pathogenic *Salmonella* strains in human disease, the impact on the incidence of human salmonellosis has been minimal [10,11]. Significantly, the *Salmonella* food safety risk begins in the intestinal tracts of poultry, primarily chickens and turkey, where these enteric organisms are important components of the intestinal microbiome. While several metabolic pathways are important for survival and growth in the intestinal environment, the type 1 fimbriae of *Salmonella* play a key role in the ability of these bacteria to persist in the intestinal microbiome of poultry organisms by mediating adherence to poultry intestinal cells [12,13,14,15,16,17]. In addition to type 1 fimbriae, many *Salmonella* serotypes encode other fimbrial gene clusters (i.e.,—Bcf, Csg, Pef, Lpf, and Tafi), although studies to identify roles for these factors in intestinal adherence have largely failed to show any significant role for these adherence factors [18]. In the absence of functional data, these putative fimbriae have no known functions, while type 1 fimbriae are essential for the establishment of *Salmonella* in the intestinal microbiome of poultry [12,13,17,18].

*Salmonella* establishes its niche in the intestinal microbiome of poultry by outcompeting other bacteria via type 1 fimbrial adherence to intestinal epithelial cells to gain access to nutrients and space [12,18,19]. Type 1 fimbriae of *Salmonella enterica* mediate binding to mannose-containing glycoconjugates on eukaryotic cell surfaces [20,21]. The fimbriae are encoded by the *fim* gene cluster and are produced on the bacterial surface using the chaperone-usher system of assembly [22]. FimA represents the major structural subunit that is polymerized to form the fimbrial shaft, and the adhesin that mediates binding to eukaryotic cells is FimH [19]. There are different alleles of the adhesin (FimH) of *Salmonella* type 1 fimbriae that possess different binding specificities for host cell receptors, with some of these alleles having significantly increased binding to host cells [12,23]. It is presumed that a FimH allele encodes for a highly adherent type 1 fimbriae that provides a competitive advantage to the *Salmonella* serotype for position and growth in the intestinal microbiome, and that different *Salmonella* serotypes have adapted their fimbrial repertoire to exploit different hosts and binding receptors [12,17,23,24].

A prospective study of *Salmonella* strains in poultry supports the idea that *Salmonella* serotypes compete to establish a presence in the intestinal microbiome [25]. This report focused on surveys of carriage of *Salmonella enterica* serovar Gallinarum and *Salmonella enterica* serovar Enteritidis in poultry in England, Wales, and Germany. *S.* Gallinarum, a host-adapted and host-restricted serovar that causes systemic disease and death in domestic and aquatic fowl, was a serious problem in the 1970s that led to a surveillance program that used “test-and-slaughter” to control *S.* Gallinarum in domestic poultry. Over time, this program led to disappearance of *S.* Gallinarum in poultry flocks and, at the same time, the emergence of the human pathogen *S.* Enteriditis as the dominant *Salmonella* serotype colonizing the domestic poultry intestinal tract, with a corresponding dramatic increase of human salmonellosis cases during this same period. The inverse relationship between these two *Salmonella* serotypes led to the conclusion that *S.* Enteriditis filled the ecological niche in the poultry intestinal tract made available by the eradication of *S.* Gallinarum from domestic poultry [25]. A conclusion of this study was that an effective way to reduce carriage of human pathogenic strains in poultry, such as *S.* Enteriditis, would be to engineer a live oral avirulent strain of *S.* Gallinarum that retained the ability to exclude *S.* Enteriditis from the poultry intestinal microbiome but that had lost its virulence for birds.

This work seeks to define the capacity of an engineered probiotic *E. coli* Nissle 1917 strain expressing hyper-adherent *Salmonella* type 1 fimbriae to selectively colonize the poultry intestinal tract and competitively exclude pathogenic *Salmonella* strains from the gut microbiome. By characterizing enhanced adherence to epithelial surfaces in both tissue culture and poultry intestinal environments, these studies establish the mechanistic foundation for development of a targeted probiotic strategy to reduce *Salmonella* colonization and transmission in commercial poultry production systems.

## 2. Materials and Methods

### 2.1. Strains, Plasmids, and Growth Conditions

*Salmonella enterica* serovar Typhimurium (*S.* Typhimurium) strains SL1344 [26] and DT104 [27] were used as representative pathogenic wild-type *Salmonella* strains and have been characterized previously. *S.* Typhimurium strain LNWI and *S.* Enteriditis NVSL 5-1952 are representative pathogenic strains that have been isolated from poultry and have been described and studied in detail previously [27]. The *S.* Typhimurium BJ3714 Δ*fimA* and *S.* Typhimurium BJ3716 Δ*hilD*-URS Δ*ssaV* strains were created using the one-step inactivation mutagenesis technique, using plasmids pKD3, pKD4, pKD46, and pCP20 [28]. Mutant strains constructed with this procedure were confirmed to have the desired mutations by PCR analysis. *E. coli* Nissle 1917 was used as the scaffold strain for the engineered probiotic strain. We will refer to *E. coli* Nissle as “*E. coli* Nissle” or “*E. coli* Nissle parent” to distinguish it from the *E. coli* Nissle engineered strain that we are characterizing here. The *fimA* and *asd* genes were deleted sequentially in *E. coli* Nissle 1917 using the one-step inactivation mutagenesis procedure [28]. The desired mutations were confirmed by PCR analysis and by functional assay for adherence or for a requirement for diaminopimelic acid (DAP) in the growth media. DAP is an essential bacterial amino acid that bacteria synthesize from precursors and is not produced by eukaryotic organisms.

Plasmid pBDJ450 was constructed by cloning partially digested (Sau3A) chromosomal DNA into pACYC184 and identifying a clone that conferred adherence onto *E. coli* HB101, which was subsequently proven to be mannose-sensitive adherence. Plasmid pBDJ450 was modified by cloning the *E. coli asd* gene onto plasmid pBDJ450. The functional *asd* gene was inserted into orf stm0551, which is a repressor of *Salmonella* type 1 fimbrial expression [29]. The inserted *asd* was shown to complement an *E. coli* strain with an *asd* deletion and the plasmid was confirmed to have intact *S.* Typhimurium type 1 fimbrial genes.

Bacterial strains were grown on L agar with appropriate antibiotics, as needed. Antibiotics were added at the following concentrations: ampicillin, 100 μg ml^ −1^; kanamycin, 25 μg ml^−1^; chloramphenicol, 25 μg ml^−1^; streptomycin 100 ug ml^−1^; tetracycline, 100 μg ml^−1^. Broth cultures of bacteria were grown statically in 10 mL of L broth with appropriate antibiotics at 37 °C and passaged after 48 h at least three times for maximal expression of type 1 fimbrial structures and binding activity.

### 2.2. Plasmid Stability Assay

To examinate the stability of plasmid pBDJ451 in its *E. coli* Nissle Δ*asd* parent strain we grew the strain in L broth with no antibiotic selection for 50 generations by passaging the strain every 12 h for 4 days. Samples were removed every 3 h and dilutions were plated on L agar plates for total number of bacteria in the culture or L agar plates with 10 μg/ml of chloramphenicol to select organisms carrying the pBDJ451 plasmid.

### 2.3. Yeast Agglutination Assay

*S. cerevisiae* yeast cells were grown with shaking overnight at 37 °C in 100 mL of medium containing peptone (2%; Difco), yeast extract (1%), and glucose (2%). The cells were harvested by centrifugation at 1500× *g* for 10 min, washed by resuspension in 100 mL of phosphate-buffered saline, pH 7.4, and sedimented again. The pellet was resuspended in 50 mL of PBS with 0.2% glutaraldehyde and incubated at room temperature for 1 h. The yeast cells were then pelleted at 1500× *g* for 10 min, washed twice with PBS, and incubated in 50 mL of PBS with 10 mg/mL of glycine for 30 min at room temperature. The fixed yeast cells were then washed again 2× in PBS and resuspended in 3 mL of PBS with 0.02% sodium azide to prevent bacterial growth, and the cells could be stored up to 1 month at 4 °C [30] (personal observations). A typical agglutination assay used 20 μL of the fixed yeast cells mixed with an equal volume (20 μL) of a suspension of a bacterial strain on a microscope slide and gently mixed to allow binding of the bacteria to the fixed yeast cells. A positive agglutination reaction gave clear clumping of the yeast cells that was mediated by binding and crosslinking via the bacteria. A negative reaction typically gave no clear clumping of yeast cells. Microscopic examination of a coverslip under low power was sometimes used to confirm agglutination reactions [30].

### 2.4. Bacterial Adherence Assay

The adherence assay has previously been described by our group [12]. Briefly, HeLa cells were maintained in RPMI 1640 tissue culture media (Gibco) with 5% fetal calf serum (FCS). Then, 10^5^ cells were seeded into each well of a 24-well tissue culture dish and incubated overnight. The following day, 1 × 10^8^ CFU of bacteria were added to each well (ratio of bacteria to cell 1000:1), and the bacteria were allowed to bind to the tissue culture cells for 3 h at 37 °C in the tissue culture incubator with gentle agitation. Following the three-hour incubation, each well was washed 3× with 1 mL of sterile PBS to remove nonadherent microorganisms or those that were loosely adherent to the tissue culture plastic. After a final wash, the cells were incubated for 10 min at room temperature in 200 μL of PBS with 0.2% Triton X-100 to solubilize the cells and release the bacteria from the membranes of the host cells, and then 800 μL of LB broth was added to each well to dilute the Triton X-100. Ten-fold dilutions from each well were then plated onto L agar with appropriate antibiotics to quantitate the number of organisms that had attached to each tissue culture monolayer over the course of the 3 h assay. Percent adherence was calculated by determining the total number of CFU recovered from each well and divided by the input number of bacteria (1 × 10^8^ CFU). Adherence for the strains is expressed either as total CFU for a given assay or as percent adherence of the input.

For the competitive adherence assays, two strains were added to the same tissue culture well at the same time. The *Salmonella* strains were selected on L strep plates, which distinguished them from the *E. coli* Nissle strain. All other steps for the adherence assay were as described above.

### 2.5. Inhibition of Type 1 Fimbrial Binding to Salmonella Rabbit Polyclonal Antibody

The inhibition of yeast agglutination by the *Salmonella* anti-type 1 fimbrial polyclonal rabbit antibody was assessed by following the protocol for the agglutination assay, but 5 μL of polyclonal antibody was added to the bacteria suspension before it was added to the yeast cells. All other procedures of the assay were the same as described previously.

### 2.6. In Vivo Competitive Exclusion Studies

Animal studies were performed at Iowa State University with approval from the Institutional Animal Care and Use Committee. For broilers and layers, one-day-old birds were obtained from Welp Hatchery, Bancroft, IA, USA. Birds were mixed genders and various breeds. Upon receiving the birds, each was checked for the presence of *Salmonella* in their feces to determine their *Salmonella* shedding status. Animal husbandry was performed as per our recent study [31]. On days 3, 4, and 5 after arrival, birds were orally inoculated with 1.2 × 10^5^ CFU of either *E. coli* Nissle 1917 pBDJ451 or *E. coli* Nissle 1917. On days 6, 7, and 8 after arrival, birds were orally inoculated with 1.2 × 10^5^ CFU of *Salmonella* Typhimurium strain LNWI [27,32]. Cloacal swabs (~1 g) were then taken from each bird every three to four days and were subjected to enumerative *Salmonella* culture using XLD agar [32]. At the end of the study, birds were euthanized with a captive bolt and ceca were harvested for enumerative *Salmonella* culture [32]. Broiler and turkey studies were performed on two separate occasions.

For layers, 20-week-old birds were housed in individual cages and procedures were performed as per the broiler studies, except that the dose of inoculated bacteria was increased ten-fold. Additionally, *S*. Enteriditis strain NVSL 5-1952 [27], which was previously isolated from poultry, was used as the test strain. For eggshell contamination studies, recovered eggs were soaked in 10 mL of LB broth, of which 100 mL was plated on XLD agar for enumeration. Layer studies were performed once using each of the 2 *Salmonella* serovars, and 20 birds were used in each group.

### 2.7. Statistics

All experiments were performed at least three separate times as independent biological replicates, and samples for a given set of experiments at the various time points were plated in triplicate. Bacterial counts were log10-transformed before analysis. Differences between two research groups were evaluated using two-tailed unpaired Student’s *t*-tests, while comparisons involving three or more groups were performed using one-way ANOVA with an appropriate multiple-comparisons test. Results are presented as mean ± standard deviation, and *p* < 0.05 was considered statistically significant.

## 3. Results

### 3.1. Construction of a Nonvirulent Salmonella Strain That Is Hyper-Adherent

**Figure 1:**
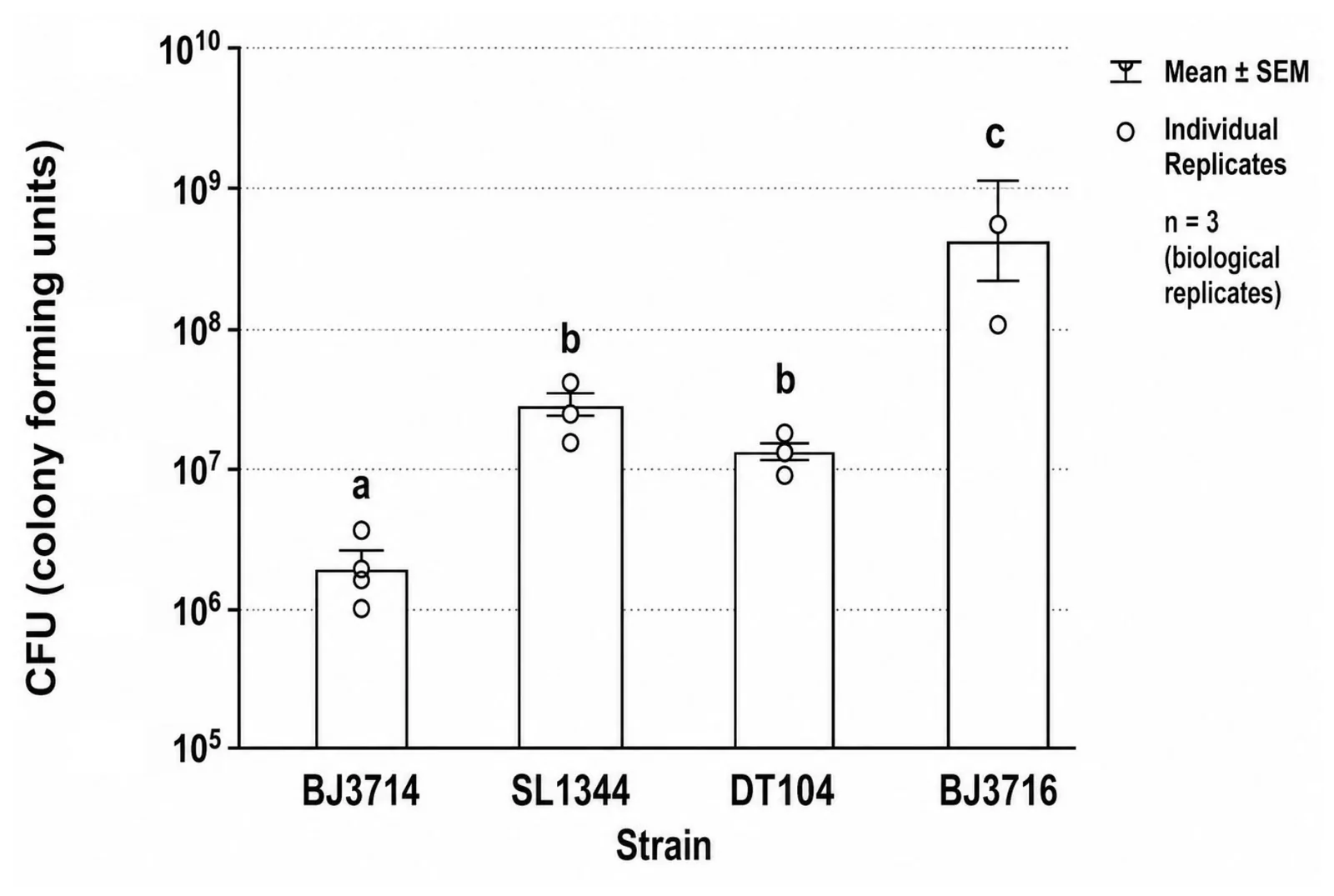
Adherence of *Salmonella* strains to HeLa tissue culture cells in vitro. Quantitated adherence is depicted in the bar graph for *S.* Typhimurium BJ3714 (*fimA*-), pathogenic *S.* Typhimurium SL1344 wild-type, multiple antibiotic-resistant *S.* Typhimurium DT104, and *S.* Typhimurium BJ3716 Δ*hilD*-URS, Δ*ssaV* hyper-adherent allele. Bacteria were grown in conditions that induce maximal expression of adherence by serially passaging cultures that were grown statically for 48 h. Then, 10^8^ CFU of each strain was added to 10^5^ HeLa cells seeded into the wells of a tissue culture dish and incubated for 3 h at 37 °C in a CO_2_ incubator. Monolayers were washed three times with PBS before lysing the monolayers with 0.2% Triton X-100 in PBS to lyse the tissue culture cells to release attached organisms, which were quantitated by diluting samples from each well and plating onto the appropriate agar plate with antibiotics and counting colonies. Four independent biological replicates of these experiments were performed, and sampling for each experiment was done in triplicate. The adherence of the strains was divided into three statistically different groups. Group a is the noninvasive BJ3714 strain, which is statistically significant from Group b, which includes SL1344 and DT104, the two wild-type *Salmonella* strains with expected adherence levels. Group c is the hyper-adherent *Salmonella* strain BJ3716, which is statistically different from all other strains. Two-sided Welch *t*-test pairwise comparisons showed that the difference in adherence of *S.* Typhimurium SL1344 vs. BJ3714 was *p* = 0.0016, and the difference in adherence of *S.* Typhimurium SL1344 vs. BJ3716 was *p* = 0.037. For comparison of *S.* Typhimurium DT104 vs. BJ3714, the difference was significant as well (*p* = 0.0028), and the difference for *S.* Typhimurium DT104 vs. BJ3716 was *p* = 0.0314.

Our first approach to create a non-pathogenic live oral probiotic strain that might have the potential to reduce *Salmonella* carriage in poultry was to engineer a non-pathogenic *Salmonella* strain that possessed high levels of T1F binding to host cells. This approach was a modification of the approach suggested by Baumler et al. [25], which was to create a live oral avirulent *S*. Gallinarum strain that maintained its ability to exclude pathogenic *Salmonella* strains. We created a noninvasive (SPI-1 mutation), intracellular-survival-defective (SPI-2 mutation) *Salmonella* strain that carries the highly adhesive type 1 fimbriae allele. This *S*. *enterica* serovar Typhimurium strain is designated as BJ3716 and carries a deletion of the *hilD* and *hilA* upstream regulatory sequences (URS), which renders *Salmonella* noninvasive [3,33,34], and the strain also carries a deletion of the s*saV* gene, which is an essential component of the type three secretion system-2 (TTSS-2) machinery [35,36,37]. Using our standard tissue culture adherence assay, the adherence of BJ3716 was compared to the adherence of pathogenic *S.* Typhimurium SL1344 and a clinical *S.* Typhimurium multiple-antibiotic-resistant DT104 isolate [27] (Figure 1). The negative adherence control for the experiments was S. Typhimurium BJ3714, which is a *fimA* isogenic mutant of BJ3716. The baseline adherence of the nonadherent negative control strain BJ3714 (T1F^−^) was 1.1 × 10^6^ CFU ± 1.2 × 10^5^ CFU, which represents about 1.1% of the original inoculum that was added to the tissue culture adherence assay well. This level of adherence in a strain that does not express type 1 fimbriae represents nonspecific adherence to the tissue culture cells and the tissue culture plastic. Pathogenic *S.* Typhimurium strain SL1344 had 1.3 × 10^7^ CFU ± 2.9 × 10^6^ CFU adherence per well and pathogenic multidrug-resistant DT104 had 9.2 × 10^6^ CFU ± 6.2 × 10^5^ CFU per well. In comparison to the negative control strain, this means that 13% of the SL1344 inoculum and 9.2% of the DT104 inoculum added to the adherence assay at the beginning of the experiment adhered in a T1F-specific manner. In direct comparison to the negative control, the two pathogenic *Salmonella* strains, SL1344 and DT104, adhere 13-fold and 9.2-fold better than the nonadherent T1F^−^ BJ3714 strain, respectively. The hyper-adherent *Salmonella* BJ3716 strain had 1.2 × 10^8^ CFU ± 6 × 10^7^ CFU adherence per well, which is 120% (or 120-fold increase over nonspecific adherence) of the original inoculum added to each well. A percentage above 100% is possible for these quantitative adherence assays since the number of CFU recovered at the end of the three-hour experiment represents both organisms in the original inoculum plus newly divided organisms that adhere to the tissue culture cells. In addition to the levels above the nonadherent strain, BJ3716 adheres 9.2-fold and 13-fold better than the two pathogenic strains SL1344 and DT104, respectively. While the *Salmonella* strain BJ3716 is both non-pathogenic and has a hyper-adherent T1F phenotype, the possibility that a *Salmonella* strain, no matter its genetic characteristics, would be approved as a viable probiotic strain for agricultural use seems unlikely. However, an important insight from this first set of experiments is that disruption of the *hilD* and the URS regulatory sequences and *ssaV* have no significant impact on the T1F hyper-adherence that we have observed in some *Salmonella* strains. These preliminary data provide impetus to our goal to create a highly adherent, yet avirulent, bacterial strain that will reduce *Salmonella* carriage in poultry. The experiments described above were repeated more than three times—each experiment was performed on a separate day, and samples were plated in triplicate for statistical significance. The differences between these strains were all highly statistically significant except when comparing *Salmonella* strains SL1344 and DT104, which both have normal adherence expected of these strains.

### 3.2. Construction of an E. coli Nissle 1917 Hyper-Adherent Probiotic Strain

Next, we engineered a probiotic in an *E. coli* Nissle 1917 scaffold strain with the highly adherent *Salmonella* T1F genes with the goal of gaining approval for the strain to be used in poultry. Construction of the probiotic strain relies on a pACYC-based plasmid that carries an LT2-derivative type 1 fimbrial gene cluster. This plasmid (pBDJ450) was modified to carry a functional *E. coli* aspartate semi-aldehyde (*asd*) gene, which provides in vivo selection of the plasmid by complementing an *asd* mutation on the *E. coli* Nissle chromosome. This plasmid, designated pBDJ451, carries a functional *Salmonella* type 1 fimbrial gene cluster and the functional *asd* gene. Since *E. coli* Nissle produces an *E. coli* version of the type 1 fimbrial gene, we deleted the *fimH* gene of the *E. coli* T1F to make the *E. coli* Nissle scaffold strain nonadherent. The phenotype of this intermediate strain was confirmed by agglutination test to be nonadherent. Since the final probiotic strain should not carry an antibiotic resistance gene, we took advantage of an in vivo selection system based upon the *asd* gene for selection of plasmid pBDJ451. The *E. coli* Nissle 1917 *asd* gene converts aspartate into diaminopimelate (DAP), an essential unique amino acid in the *E. coli* bacterial cell wall that cannot be scavenged from eukaryotic organisms. The chromosomal *asd* gene was deleted using the one-step inactivation procedure [28], which replaced the functional *asd* gene with a chloramphenicol resistance gene, flanked by FRT sequences. The chloramphenicol resistance gene was subsequently deleted using the Flp recombinase on the temperature-sensitive plasmid pCP20, which created an *E. coli* Nissle Δ*asd* T1F^−^ mutant strain. The mutant was unable to grow without the addition of DAP to the growth media, but when pBDJ451 was transformed into *E. coli* Nissle 1917 Δ*asd* the strain grew as well as wild-type *E. coli* Nissle without the addition of DAP, indicating that the in vivo selection proceeded as expected. The engineered *E. coli* Nissle pBDJ451 probiotic strain is an avirulent strain that should have most, if not all, of the same beneficial properties of the *E. coli* Nissle parent strain. Those beneficial properties include a strong intestinal colonization capacity, nutrient and iron competition with pathogens, microcin production, enhancement of epithelial barrier integrity, and modulation of mucosal immune responses (reference). The only significant change in the *E. coli* Nissle pBDJ451 probiotic from its Nissle parent is that the probiotic expresses a *Salmonella* type 1 fimbriae and the parent expresses an *E. coli* type 1 fimbriae.

We have also performed experiments with the *E. coli* Nissle pBDJ451 probiotic strain to examine the stability of the plasmid in the absence of antibiotic selection. A starter culture was grown in L cam, which ensures that all organisms carried the pBDJ451, which is camR. Subsequently, the culture was put into a 100 mL flask of L broth with no antibiotic and grown for 15 h. After 15 h, a second 100 mL flask was inoculated from the first culture and the culture was grown, without antibiotics, for an additional 10 h. Cultures were removed at 0, 5, 10, 15, and 25 h and appropriately diluted before plating on L agar and L agar plates with 10 μg/ml of chloramphenicol. Bacteria growing on L agar plates gave the total CFU present in the culture, and bacteria growing on L agar with 10 μg/ml of chloramphenicol were CFU that retained the pBDJ451 plasmid. The ratios of bacteria growing on L agar vs. L agar cam^10^ were 1:1.03 ± 0.13 at hour 0, 1:1.00 ± 0.13 at hour 5 (10 generations), 1:1.02 ± 0.14 at hour 10 (20 generations), 1:0.98 ± 0.13 at hour 15 (30 generations), and 1:0.94 ± 0.13 at hour 25 (50 generations). Three biological replicates of this experiment were performed, and each time point in each experiment had three separate platings to determine CFU. Standard deviations for each time point were determined from all nine data points for each experiment. As shown, the pBDJ451 plasmid was highly stable in the E. *coli* Nissle Δ*asd* host strain, as expected since the strain is unable to grow without the functional *asd* gene encoded on pBDJ451. While we have not tested the transmissibility of this plasmid, it is likely to be very difficult to transfer to other bacteria since it has no conjugation genes nor any other known genes involved in transmission.

### 3.3. Adherence Phenotypes of E. coli Nissle 1917 pBDJ451

**Figure 2:**
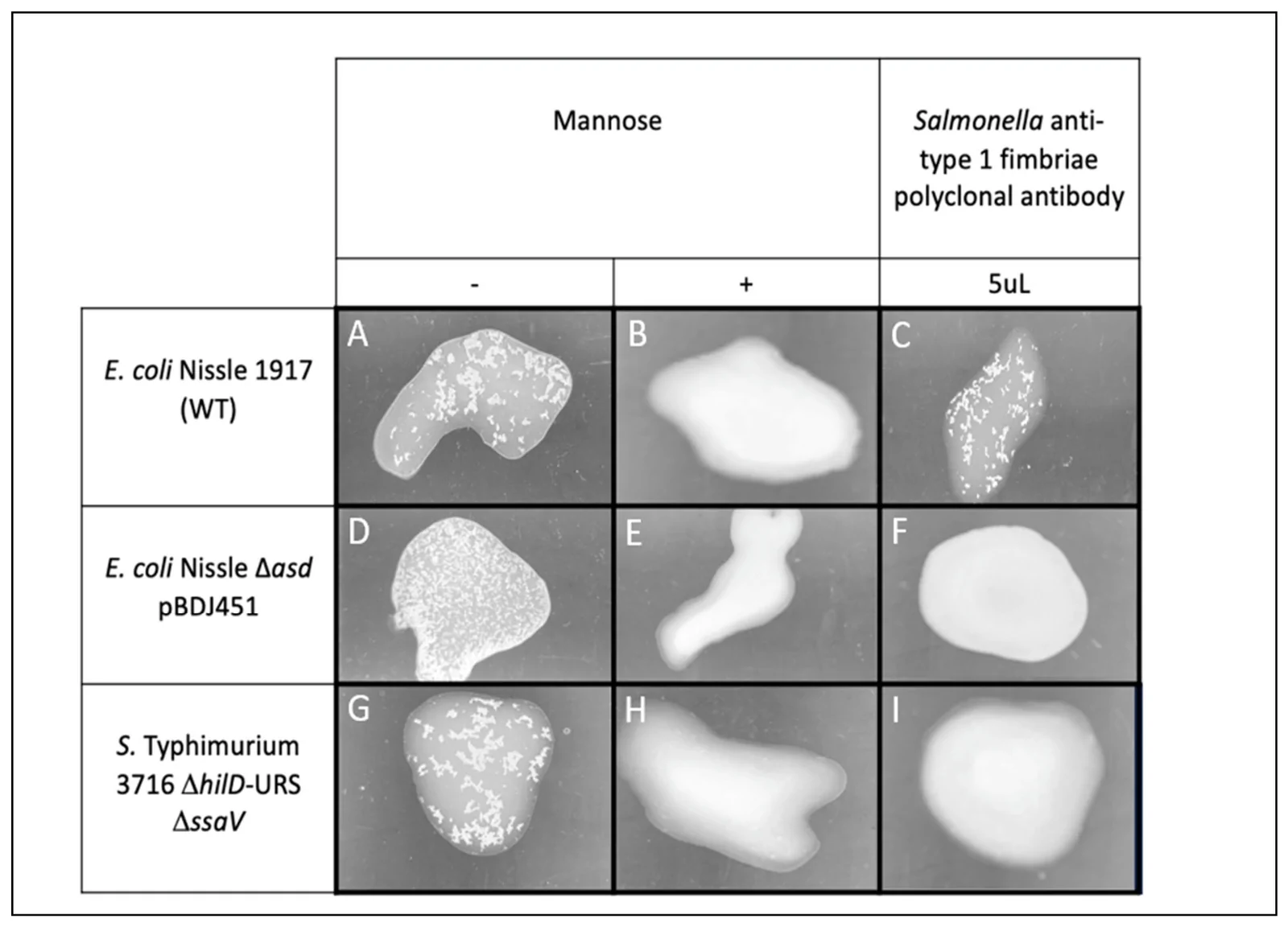
Agglutination phenotype of the *E. coli* Nissle 1917 pBDJ451 probiotic strain. The *E. coli* Nissle pBDJ451 probiotic strain, the *E. coli* Nissle 1917 control, and the *S.* Typhimurium BJ3716 control were grown in adherence-inducing conditions. Typically, yeast cells were prepared fresh on the day of the assay, and 20 μL of yeast cells were mixed with 20 μL of 10-fold concentrated bacterial cultures and deposited on a clean microscope slide. The mixture was gently mixed for 2–5 min until the positive control strain was agglutinated. As seen in panels (**A**,**D**,**G**), each of the strains clumped dramatically, indicating a positive reaction. In all instances, 1 mM mannose inhibited the agglutination observed in the absence of mannose (panels (**B**,**E**,**H**)). *Salmonella* anti-type 1 rabbit polyclonal antibody specifically inhibits *Salmonella* type 1 fimbriae agglutination activity, as seen in panels (**F**,**I**), but it does not inhibit *E. coli* type 1 fimbrial agglutinating activity, as seen in panel (**C**).

After construction of the hyper-adherent probiotic strain, we confirmed that *E. coli* Nissle 1917 Δ*asd* pBDJ451 expressed mannose-sensitive *Salmonella* type 1 fimbriae using a standard agglutination assay. *E. coli* Nissle 1917, *E. coli* Nissle Δ*asd* pBDJ451, and *S.* Typhimurium BJ3716 were grown in conditions that induce expression of type 1 fimbriae. Each of the three strains were tested for agglutination in the presence or absence of mannose or in the presence of anti-*Salmonella* type 1 fimbriae antibody, which inhibits *Salmonella* type 1 fimbriae-mediated agglutination by binding to the fimbria and preventing them from agglutinating [38,39]. As seen in Figure 2A,B, *E. coli* Nissle 1917 agglutinates yeast cells in a mannose-sensitive manner, but the agglutination mediated by the type 1 fimbriae produced by *E. coli* Nissle 1917 is not blocked by the anti-*Salmonella* type 1 fimbriae antibodies (Figure 2C), indicating that the agglutination observed for *E. coli* Nissle 1917 wild-type is mediated by *E. coli* type 1 fimbriae. Similarly, the hyper-adherent *Salmonella* BJ3716 strain agglutinates yeast cells in a mannose-sensitive manner (Figure 2G,H) but, in contrast to *E. coli* Nissle agglutination, is blocked by the anti-*Salmonella* type 1 fimbriae antibodies (Figure 2I). The probiotic *E. coli* Nissle Δ*asd* pBDJ451 also displayed a strong yeast agglutination phenotype that was blocked by mannose (Figure 2D,E) but, importantly, the *Salmonella* anti-type 1 fimbriae antibodies inhibited the agglutination reaction (Figure 2F), confirming that the *E. coli* Nissle Δ*asd* pBDJ451 strain produces significant amounts of *Salmonella* type 1 fimbriae that bind to eukaryotic cells in a mannose-sensitive manner.

**Figure 3:**
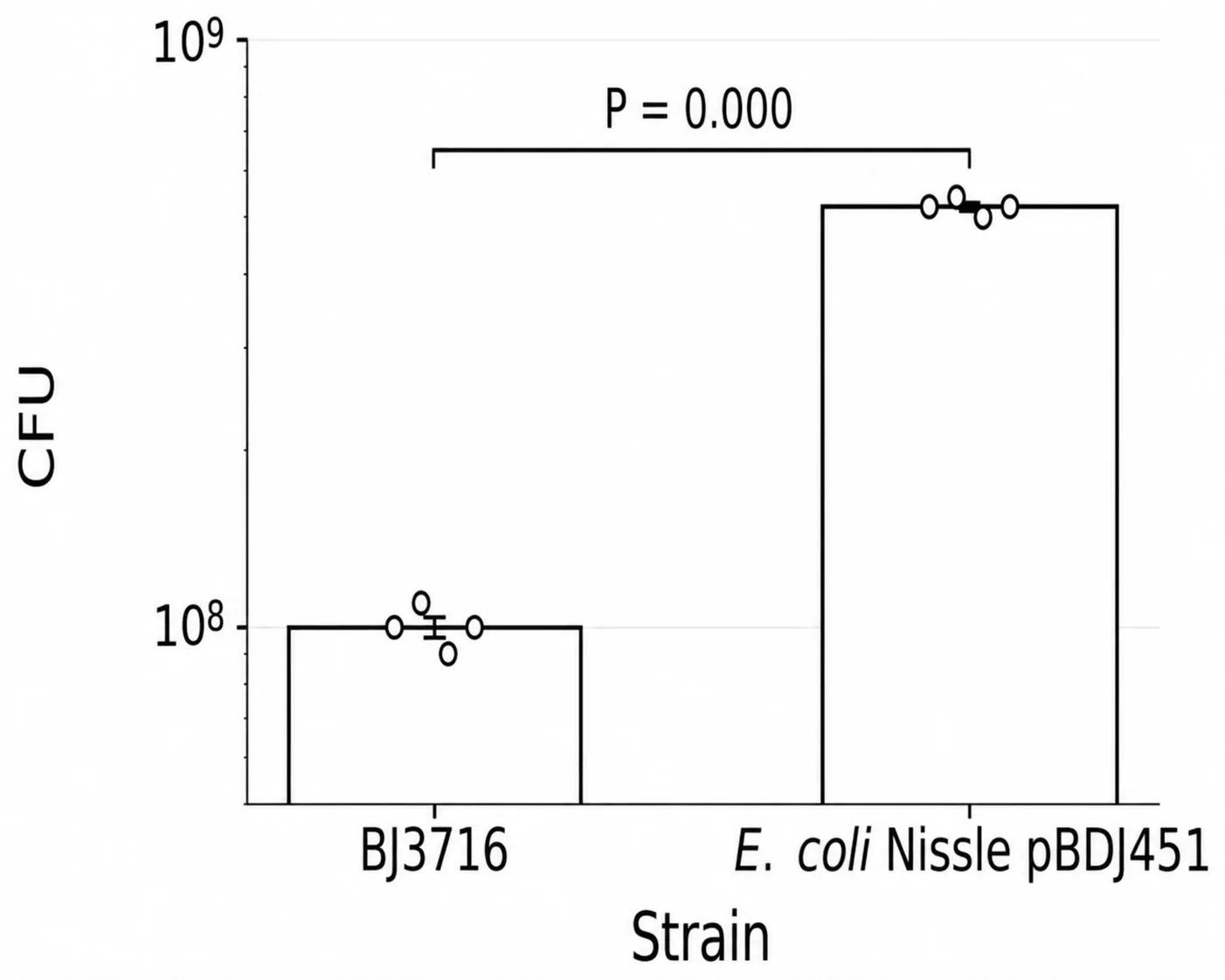
Adherence of hyper-adherent *S.* Typhimurium BJ3716 compared to *E. coli* Nissle 1917 pBDJ451. Adherence of the *E. coli* Nissle 1917 pBDJ451 was quantitated using the standard adherence assay and compared to hyper-adherence observed for BJ3716. The adherence observed in the *E. coli* probiotic strain was ~5-fold higher than observed for BJ3716. Four independent biological replicates of this experiment were performed with sampling for individual experiments done in triplicate. One-way ANOVA was performed on the data for statistical analysis. The data points are shown with the standard deviation bars, and the *p*-value was 0.0004.

We previously demonstrated that *S*. Typhimurium strain BJ3716 possessed adherence that was 10-fold higher than either *S*. Typhimurium SL1344 or *S.* Typhimurium DT104 in tissue culture adherence assays (Figure 1). While this difference in adherence may not seem important, it is 10-fold higher than fully adherent strains of pathogenic Salmonella, leading us to designate this adherence “hyper-adherence”. We wanted to quantitate adherence produced by *E. coli* Nissle 1917 Δ*asd* pBDJ451 and compare it to adherence observed with *S.* Typhimurium strain BJ3716. In a comparative tissue culture adherence assay, we observed 1.0 × 10^8^ CFU ± 4 × 10^7^ CFU adherence for *S.* Typhimurium BJ3716 and 5.2 × 10^8^ CFU ± 9 × 10^7^ CFU for *E. coli* Nissle 1917 Δ*asd* pBDJ451 (Figure 3). Thus, the *E. coli* Nissle 1917 pBDJ451 probiotic strain displayed even higher adherence (5.2-fold higher) than the hyper-adherent *S*. Typhimurium strain BJ3716. This surprising result is likely due to *Salmonella* type 1 fimbrial proteins being produced by multicopy plasmid pBDJ451 and bodes well for the ability of this strain to outcompete pathogenic *Salmonella* strains in binding to host cells in the intestinal environment. These experiments were repeated three times, each experiment being conducted on a separate day, and samples were plated in triplicate for statistical significance.

### 3.4. Competitive Adherence Assay as a Model for Competitive Exclusion

**Figure 4:**
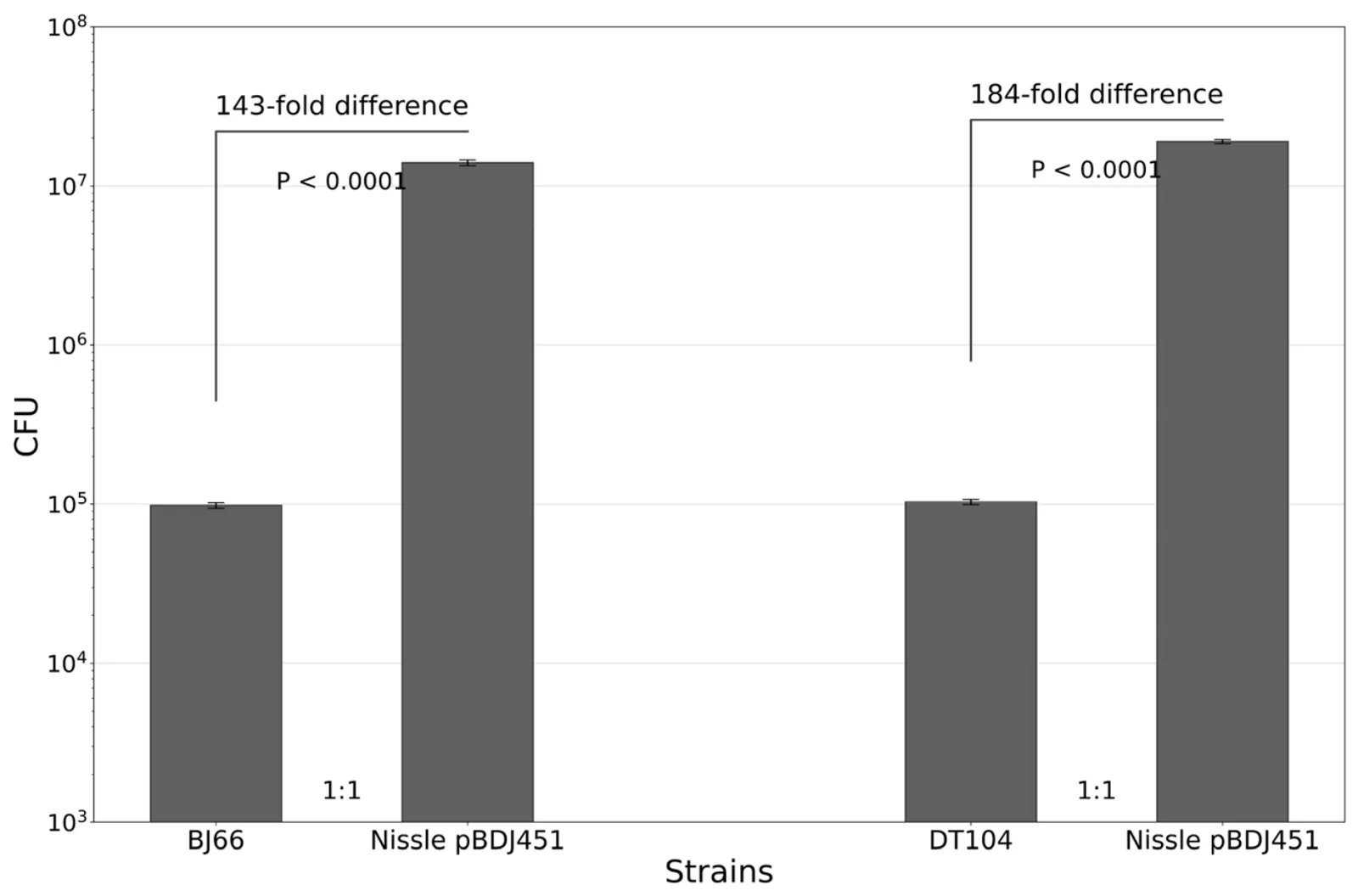
Competitive adherence assay examining the adherence of the *E. coli* Nissle pBDJ451 probiotic strain in the presence of pathogenic *Salmonella* strains. The ability of strains to competitively adhere to tissue culture cells was measured in a standard adherence assay. In one set of experiments, the binding of *E. coli* Nissle pBDJ451 was compared to *S.* Typhimurium SL1344 binding (left-side bars), and the other comparison was *E. coli* Nissle 1917 pBDJ451 compared to *S.* Typhimurium DT104 (right-side bars). Each strain was grown in conditions that induce the adherence phenotype. Then, 10^8^ CFU of each strain was added to tissue culture wells seeded the previous day with 10^5^ HeLa cells and adherence to the cells was allowed to proceed for 3 h. Adhered bacteria were washed three times with PBS, the cell monolayers were lysed with 0.2% Triton X-100 in PBS, diluted with L broth, and then dilutions from each well were plated on agar plates with selective antibiotics for each strain. Four independent biological replicates of this experiment were performed with sampling from individual experiments performed in triplicate. The data shown in the figure are a representation of the average of those results. One-way ANOVA was performed on the data to determine statistical significance. Fold differences and *p*-values are shown in the figure.

We demonstrated that the engineered *E. coli* Nissle 1917 Δ*asd* pBDJ451 recombinant strain possesses the ability to bind to tissue culture cells at high levels, which is ~500-fold higher than nonadherent *Salmonella* strain BJ3714 *fimA* mutant, 20–50-fold higher adherence than pathogenic *Salmonella* strains SL1344 and DT104, respectively (Figure 1), and even ~5-fold higher than engineered hyper-adherent *Salmonella* strain BJ3716 (Figure 3). We wanted to assess whether there is a difference in binding activity when two strains were competing to adhere to the same tissue culture monolayer. We developed this assay as a model to assess one aspect (adherence) of the ability of the *E. coli* Nissle 1917 pBDJ451 probiotic strain to competitively exclude pathogenic *Salmonella* strains from binding to host cells. Adherence assays were performed as described for single strain adherence assays with the exception that both strains (1 × 10^8^ CFU/well) were added to the well at the same time and allowed to bind to the tissue culture monolayer. As shown in Figure 4 (left-side bars), the hyper-adherent *E. coli* Nissle pBDJ451 probiotic strain adhered 143× higher to tissue culture cells when competing with *Salmonella* SL1344 and 185× higher when competing with *Salmonella* DT104 for adherence (Figure 4, right-side bars). These results are very encouraging and suggest that the *E. coli* Nissle 1917 pBDJ451 has the potential to adhere at very high levels to chicken intestinal epithelial cells and exclude pathogenic *Salmonella* strains from adhering at levels that are typical for *Salmonella* when colonizing the chicken intestinal tract. The competition assays were repeated a total of three times on three separate days, with the data shown in Figure 4 representative of each of the experiments. Samples were plated in triplicate for each individual experiment as well to provide statistical significance.

### 3.5. E. coli Nissle 1917 pBDJ451 Competitively Excludes Salmonella in Poultry

**Figure 5:**
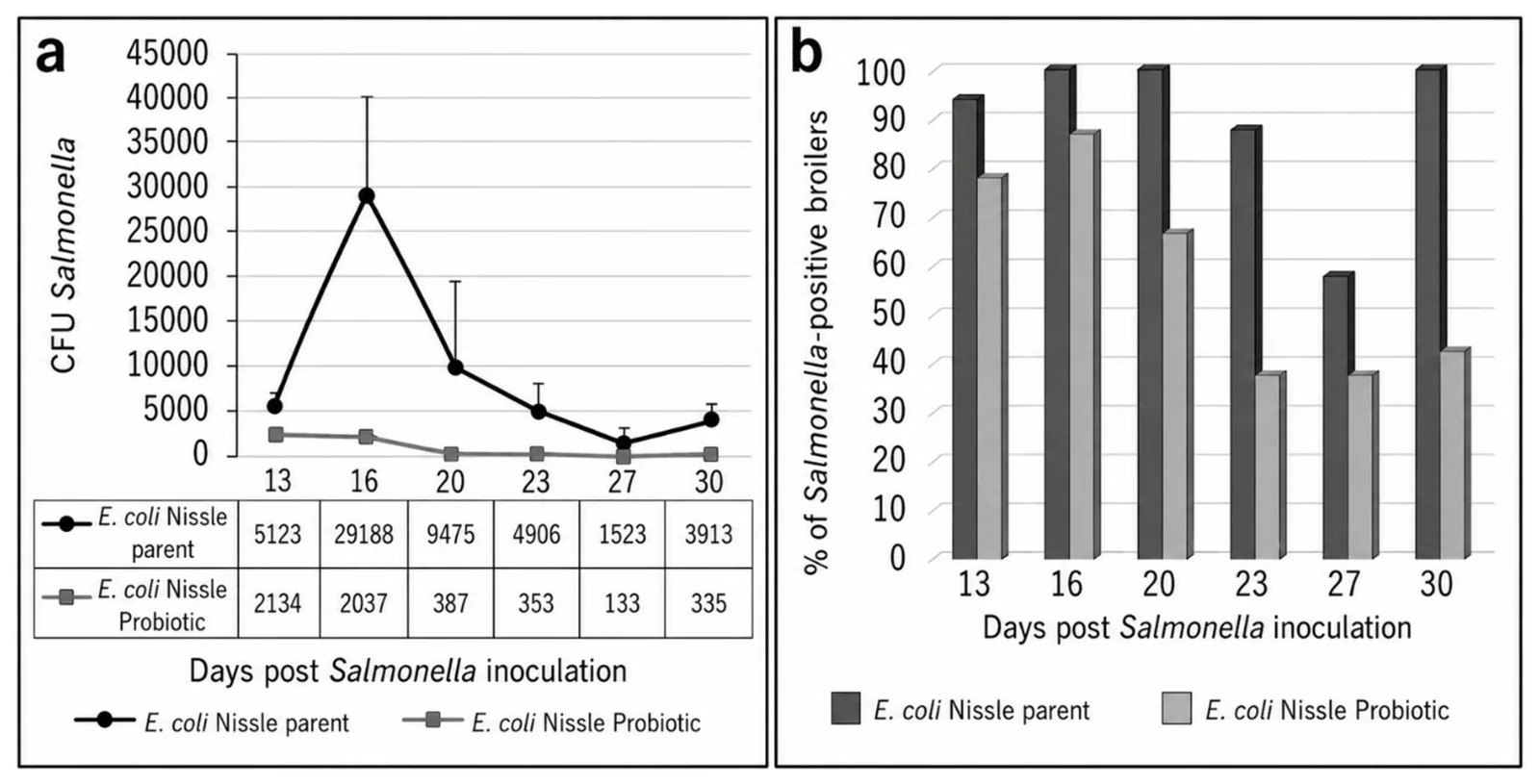
Effect of *E. coli* Nissle pBDJ451 probiotic on *Salmonella* carriage in chicken broilers. Groups of 20 broiler chicks were inoculated with either 10^5^ CFU of *E. coli* Nissle 1917 (parental control) or 10^5^ of the *E. coli* probiotic strain that carries *Salmonella* hyper-adherence genes. Two days later, each chick received 10^5^ CFU of *Salmonella* Typhimurium. The carriage and persistence of *Salmonella* in each of the chicks was followed by collecting cloacal swabs from each bird and plating to quantitate the *Salmonella* Typhimurium CFU in each bird. Shown in (**a**) are the *Salmonella* CFU enumerated from each group of birds on days 13, 16, 20, 23, 27, and 30 post-colonization. The data shown with the black line are broiler chicks receiving the *E. coli* Nissle parental control and those with the gray line are the birds receiving the *E. coli Salmonella*-specific probiotic strain. (**b**) Percentage of birds in each group that had any *Salmonella* isolated from cloacal swabs on the day indicated.

To determine if in vitro hyper-adherence leads to competitive exclusion of wild-type *Salmonella* from the intestines of poultry, we performed competitive exclusion experiments in both chicken broilers and turkeys. Two separate chicken broiler competitive exclusion experiments were performed in which *E. coli* Nissle 1917 pBDJ451 was orally inoculated into one-day-old broiler chicks for three consecutive days. On the fourth and fifth days, birds were orally inoculated with 10^5^ CFU of wild-type *Salmonella* Typhimurium and then cloacal swabs were analyzed for *Salmonella* shedding. Cloacal swabs were collected every 3–4 days and swabs were subjected to enumerative *Salmonella* culture. At the end of the study birds were euthanized and ceca were harvested and subjected to enumerative *Salmonella* culture. As a control, a parallel cohort of birds were orally inoculated with parent *E. coli* Nissle 1917. As seen in Figure 5a, at the earliest point there was only a 2-fold difference in *Salmonella* carriage in Nissle parent-colonized birds and Nissle probiotic-colonized birds, but that difference changed dramatically over the course of the experiment. The number of CFU of *Salmonella* isolated from Nissle parent-colonized birds varied from about 4000 to 5000 (calculated from dilution plates) at each sampling, while the Nissle probiotic-colonized birds had about 300–400 CFU of *Salmonella* recovered, which is about a 12–16-fold reduction (>1 log reduction, which is also a 90% reduction in counts) of *Salmonella* carriage in Nissle probiotic-treated broilers. The incidence of *Salmonella* carriage in broilers is also favorably altered by the Nissle probiotic in that a higher percentage of birds had undetectable levels of *Salmonella* in their feces (Figure 5b). These trends were observed in both of the broiler experiments that we performed, although the trending differences were not statistically different.

**Figure 6:**
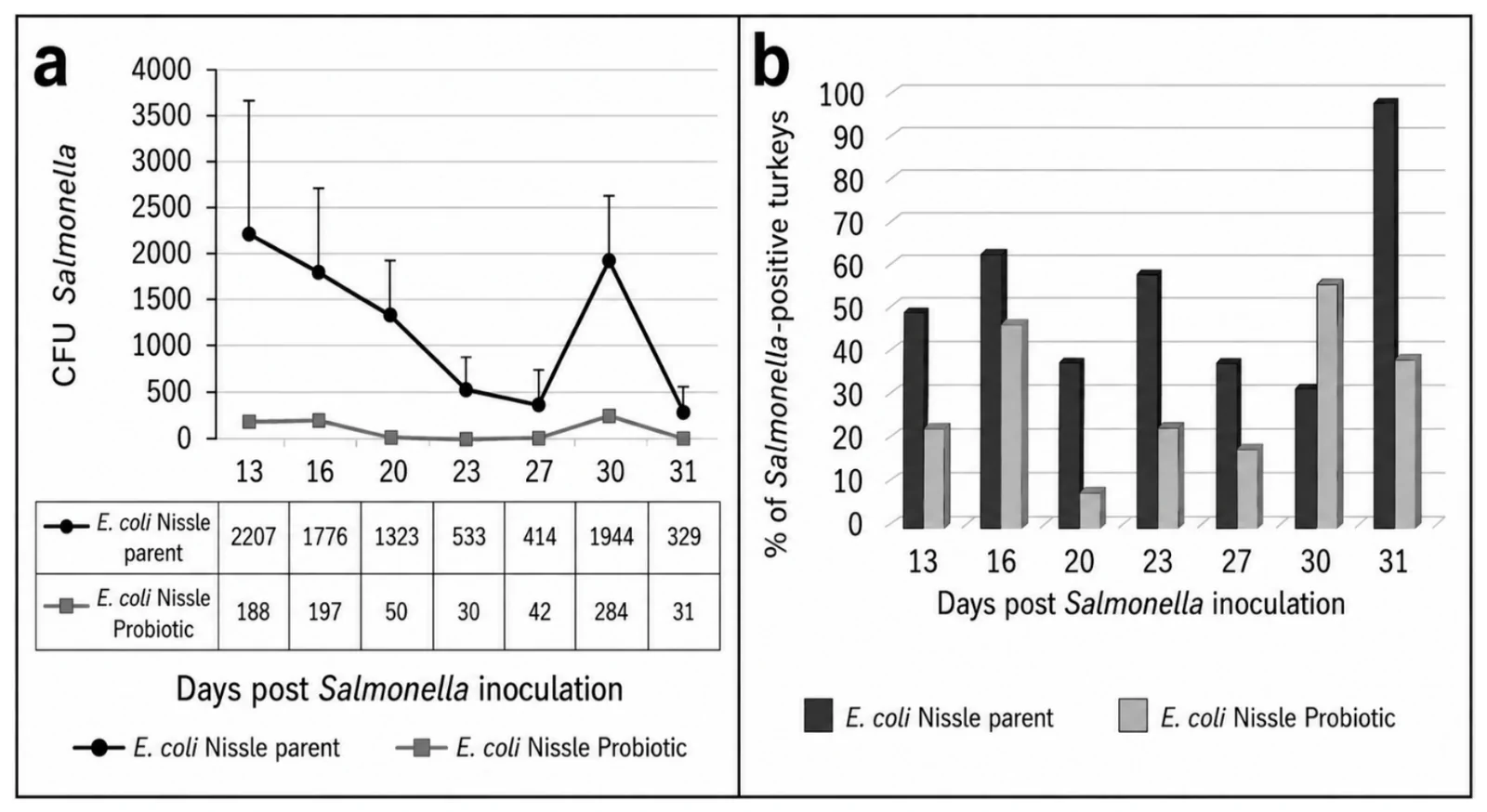
Effect of *E. coli* Nissle pBDJ451 probiotic on *Salmonella* carriage in turkey poults. Groups of 20 turkey poults were inoculated with either 10^5^ CFU of *E. coli* Nissle 1917 (parental control) or 10^5^ of the *E. coli* probiotic strain that carries *Salmonella* hyper-adherence genes. Two days later, each turkey poult received 10^5^ CFU of *Salmonella* Typhimurium. The carriage and persistence of *Salmonella* in each of the poults was followed by taking cloacal swabs from each bird and plating to quantitate the *Salmonella* Typhimurium CFU in each bird. Shown in (**a**) are the *Salmonella* CFU collected from each group of birds on days 13, 16, 20, 23, 27, and 30. The data shown with the black line are turkey poults receiving the *E. coli* Nissle parental control and those with the gray line are the birds receiving the *E. coli Salmonella*-specific probiotic strain. (**b**) Percentage of birds in each group that had any *Salmonella* isolated from cloacal samples on the day indicated.

Two separate turkey colonization experiments were also performed with the *E. coli* Nissle probiotic strain. An identical inoculation and sampling protocol was followed as for the chicken broilers—*E. coli* Nissle 1917 pBDJ451 was orally inoculated into one-day-old turkey poults for three consecutive days. On the fourth and fifth days, birds were orally inoculated with wild-type *Salmonella* Typhimurium and cloacal swabs were analyzed for *Salmonella* shedding. Samples were collected every 3–4 days and swabs were subjected to enumerative *Salmonella* culture. As seen in Figure 6a, there is a dramatic reduction of carriage of *Salmonella* in turkey poults in birds receiving the Nissle probiotic vs. birds receiving the Nissle parent strain. For the Nissle parent-treated birds, colony counts dropped from several thousand organisms early during the experiment to 300–400 CFU as time went by, while the probiotic-treated birds started at about 200 CFU of *Salmonella* and those numbers dropped to about 30 CFU during the later stages of the competitive exclusion experiment. There was typically about a 10–20-fold difference (>1 log reduction, which is also a 90% reduction in counts) in *Salmonella* carriage between the Nissle and probiotic-treated birds. The reduction in *Salmonella* carriage extended to the incidence of birds even positive for *Salmonella* carriage, as observed in Figure 6b. As can be observed, birds receiving the Nissle probiotic strain were significantly less likely (50% reduction or more) to even be positive for *Salmonella* in cloacal samples, although the incidence data at day 30 seemed to be an outlier. These trends were observed in both of the turkey experiments that we performed, although the trending differences were not statistically different. There was no obvious reason why the carriage of *Salmonella* in turkeys receiving the Nissle parent strain should increase dramatically on day 30. These data are highly promising for reducing *Salmonella* carriage in turkeys, as well as chicken broilers (Figure 5).

**Figure 7:**
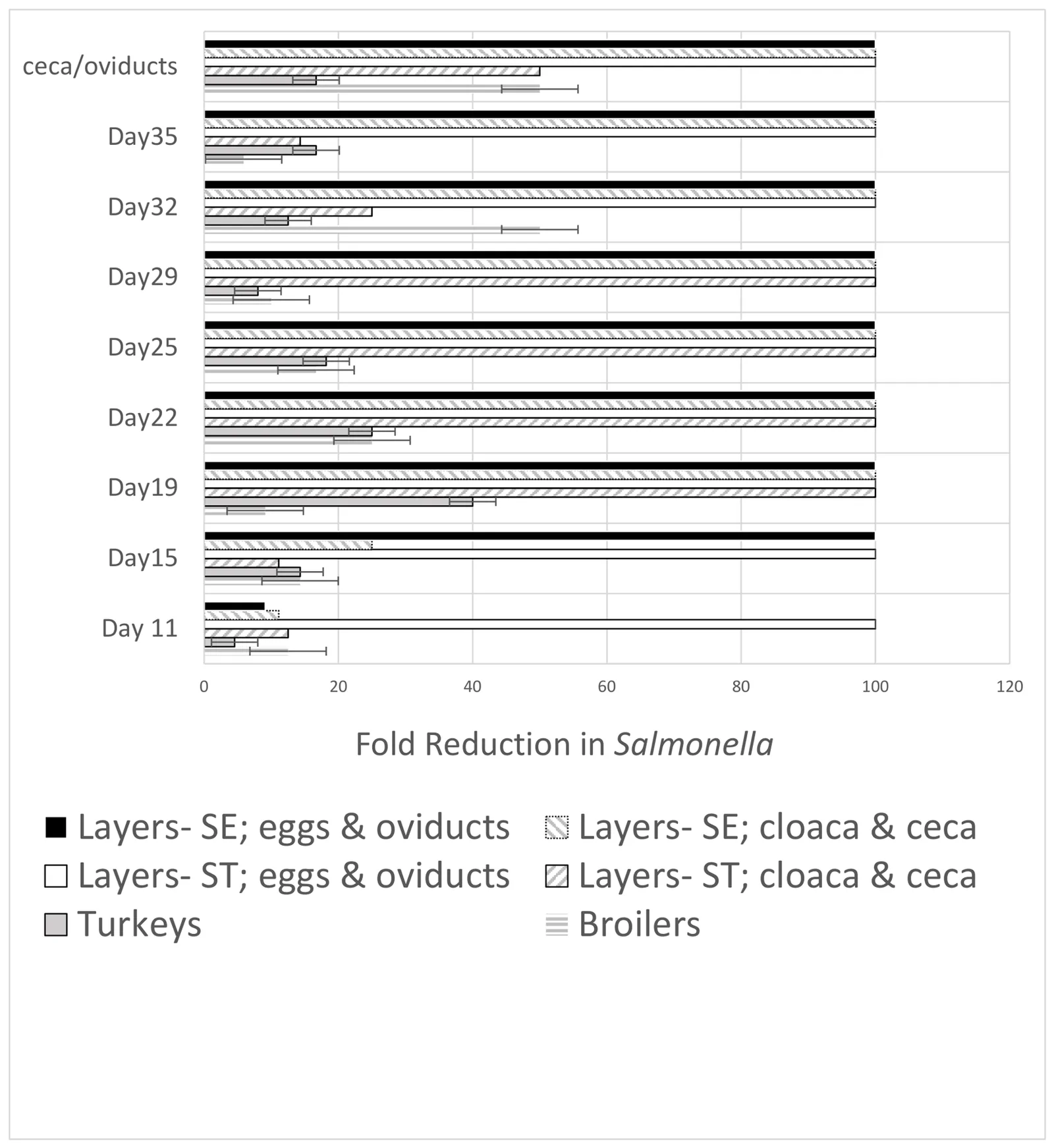
In vivo reduction of cloacal shedding, cecal colonization, egg contamination, and oviduct colonization of experimentally introduced *Salmonella* in poultry inoculated with *E. coli* Nissle 1917 pBDJ451. Two experiments were performed with chicken layers, as described for broilers in Figure 5 and turkeys in Figure 6. In one of the layer experiments, the layers were challenged with *S*. Typhimurium, and in the other experiment they were challenged with *S*. Enteriditis. Samples were taken of cloaca and ceca and of eggs and oviducts to screen for the presence of *Salmonella* in each. As described for broilers and turkeys in the experiments above, layers were orally inoculated with *E. coli* Nissle 1917 pBDJ451 followed by oral inoculation with *Salmonella*. *Salmonella* burden (cloacal shedding for all birds and egg contamination for layers) was measured every three to four days on eight occasions. At the end of the study, birds were euthanized, and ceca were harvested from all birds and oviducts were harvested from layers for enumerative *Salmonella* culture. All three types of birds were inoculated with *S*. Typhimurium (ST), while one layer experiment involved inoculation with *S*. Enteriditis 5-1952 (SE). As a control, birds were orally inoculated with *E. coli* Nissle 1917 and fold reduction of *Salmonella* was calculated based on the presence of *Salmonella* in the control birds, whereby a value of 100 indicates complete elimination of the *Salmonella*. Data represent the mean ± SEM for 20 birds per treatment group. Data presented are the compilation of the above.

Figure 7 is a compilation of data to compare broilers treated with the probiotic, turkeys treated with the probiotic, and chicken layers treated with the probiotic also. As can be seen, shedding and cecal colonization of *Salmonella* Typhimurium was reduced from 10–100-fold (90–99% reduction) in broilers, turkeys, and layers inoculated with *E. coli* Nissle 1917 pBDJ451. *Salmonella* eggshell contamination and ascending colonization of the oviduct was also significantly reduced in layers orally inoculated with *E. coli* Nissle 1917 pBDJ451. This reduction in *Salmonella* burden was also observed for layers orally inoculated with *Salmonella* Enteriditis 5-1952. In Figure 7, 40 birds are represented as colonized with parent *E. coli* Nissle or *E. coli* Nissle probiotic for broilers, for turkeys, or for chicken layers. Layers used were also one-day-old birds.

## 4. Discussion

The Centers for Disease Control and Prevention estimate that *Salmonella* species cause an estimated 1.35 million cases and 26,500 hospitalizations per year in the United States [40]. According to WHO assessments, the worldwide cases of *Salmonella* gastroenteritis approach 90 million per year with about 150,000 associated deaths, making it one of the most important foodborne infections. More than 30% of these cases are the result of consumption of poultry that is contaminated with pathogenic *Salmonella* species. Chicken and turkey carcasses are commonly contaminated with *Salmonella* species, as well as other intestinal organisms, and fecal contamination of eggs can also be a major source of microbiologic contamination. *S.* Typhimurium and *S.* Enteritidis are commonly isolated serovars isolated from contaminated chicken and often cause symptomatic clinical disease. While the food safety problems associated with *Salmonella* carriage in poultry are well understood because of extensive research efforts, providing solutions to the issue have remained an ongoing effort. A variety of approaches are being used to reduce the risk of *Salmonella* carriage in poultry that include the use of prebiotics, probiotics, bacterial subproducts, bacteriophage therapy, and vaccines that provide *Salmonella* control [41]. Some of these approaches are promising, but each has various difficulties or shortcomings that need to be addressed.

The probiotic approach described in this study seeks to take advantage of *Salmonella* adherence factors that are crucial to the ability of the bacteria to colonize the poultry intestinal tract. Research efforts, by many groups, seeking to identify and characterize the key factors that allow *Salmonella* to establish colonization in poultry, have led to the general conclusion that type 1 fimbriae are a key factor in colonizing the poultry intestinal tract [12,13,17,18]. A variety of studies have characterized the genetics, expression, and functions of these organelles [14,19,22,42,43,44,45]. Other work has explored the host immune response to *Salmonella* fimbriae. Efforts have been focused on determining if host immunity produces a neutralizing response to fimbrial structures so that vaccines might be developed to disrupt their ability to reduce or eliminate *Salmonella* colonization [46,47,48,49]. While there are a variety of immunological responses to the fimbriae in the host, the responses that are directed against intra-intestinal organisms appear to be ineffective and to date are unable to prevent the organism from colonizing the intestinal tract of the host [50,51].

A wide variety of different factors play a role in the ability of *Salmonella* to establish itself as a long-term resident of the intestinal microbiome in poultry. Factors that impact the ability of the bacteria to become a microbiome resident include access to host cell receptors for anchoring of the organisms to the epithelium of the gut, competition for nutrients with other organisms in the microbiota for growth, and the effects of inhibitory substances (i.e., bacteriocins, secondary metabolites, etc.) that are produced in the intestinal that inhibit growth of, or kill, *Salmonella*. Collectively, these factors are termed the “microbial barrier” that provides colonization resistance to organisms that may be pathogenic or deleterious to the host [52,53,54]. A prime example of the importance of the microbial barrier is observed when antibiotic treatment destroys the intestinal microbiome, allowing *Clostridium difficile* an opportunity to cause intestinal colitis [55]. Similarly, infection experiments performed in germ-free animals provide strong evidence that the ability of a pathogen to cause disease is significantly reduced by the presence of an intact microbiome [56,57].

A large number of preharvest interventions have been studied in efforts to reduce *Salmonella* prevalence and levels as the poultry enter slaughter and processing. These interventions include feed/water additives, competitive exclusion cultures, vaccination/immunization, chemical treatments of compounds, and probiotics. A systematic review of these studies has provided a broad overview of the published evidence of these interventions, including probiotic approaches that impact preharvest levels of *Salmonella* [58]. Two probiotic studies have shown particular effectiveness in reducing Salmonella carriage. Knap et al. [59] evaluated dietary supplementation with *Bacillus subtilis* DSM17299 in broilers under production-like conditions and reported a significant reduction in mean cecal *Salmonella* loads and a 58% reduction in *Salmonella*-positive environmental drag swabs. The authors in this study delivered the probiotic *B. subtilis* strain each day of the experiment and, since this strain favors the growth of lactic-acid-producing bacteria, the mechanism is believed to be creating an acidic environment in the intestinal tract. These findings demonstrate that a selected *Bacillus* strain can substantially reduce intestinal colonization and environmental contamination, although improvements in growth and feed conversion were not statistically significant. Carter et al. [60] evaluated combined administration of *Lactobacillus salivarius* [59] and *Enterococcus faecium* PXN33 using direct-challenge and bird-to-bird transmission models. The combination produced a significant 2-log_10_ reduction in *S. Enteritidis* colonization in the ileum, cecum, and colon at day 43, whereas neither organism alone reduced colonization of *Salmonella*. Administration timing influenced protection in the transmission model, highlighting the importance of both strain combinations and treatment schedules for effective probiotic control of *Salmonella*. This work reports the characterization of an engineered probiotic derived from *E. coli* Nissle 1917 that expresses hyper-adherence mediated by type 1 fimbriae (T1F) and displayed the ability to reduce *Salmonella* carriage in birds colonized with the *E. coli* Nissle pBDJ451 probiotic strain when compared to the parent *E. coli* Nissle strain. Our favored explanation for this competitive exclusion is that the probiotic strain functions by outcompeting for binding the pathogenic *Salmonella* strains. We believe that the in vitro data presented in this manuscript strongly support this explanation but there are alternative explanations for these results. Two other possibilities are that (1) the probiotic strain alters the microbiota composition to cause exclusion of the *Salmonella* strains, or that (2) the probiotic strain is significantly better at nutrient competition than the *Salmonella* strains. For both of these explanations, it would be necessary for the *E. coli* Nissle probiotic strain to have a significant impact on the microbiota composition or to be better at nutrient competition than the parent *E. coli* Nissle strain since the differences that we observed are compared to birds receiving the *E. coli* Nissle parent strain as the control treatment. Future work will focus on establishing which, if any, of these mechanisms are responsible for the carriage reductions observed. In addition, it is important to note that in this work we only examined the ability of the *E. coli* probiotic strain to competitively exclude one *S.* Typhimurium strain and one *S.* Enteriditis strain from poultry intestinal colonization. Future work will also focus on determining whether the *E. coli* Nissle strain persists for a long period of time in the intestinal tract. While we believe that the reduction of *Salmonella* carriage that we observed with these two strains is promising because of the level of reduction, we acknowledge that the ability of the *E. coli* Nissle probiotic to reduce colonization of other pathogenic *Salmonella* strains and serovars needs to be examined in detail before the value of this approach can be fully analyzed.

Unlike conventional antimicrobial strategies that attempt to eliminate pathogens after colonization has occurred, this approach is designed to occupy the ecological niche required for *Salmonella* persistence before pathogenic strains can establish themselves within the intestinal microbiome. The historical replacement of *S. Gallinarum* by *S. Enteriditis* poultry production systems demonstrates that superior intestinal competitors can profoundly shape pathogen ecology and disease emergence. In order for this work to be applicable to commercial poultry production, it will be important to optimize the dosage of *E. coli* Nissle probiotic that can be delivered to the birds and to establish that the probiotic strain can colonize and exert a long-term influence on exposure to *Salmonella* from the environment. In addition, it is important to demonstrate that the strain has no adverse effects on bird health and/or performance and that it has no negative activity in the commercial poultry environment. Critically, these early results will also need to be extended to field-relevant conditions. An important consideration is that even if highly effective, the probiotic may be introduced into the birds after *Salmonella* has already colonized the intestinal tract. Because of the high affinity for binding to receptors in the intestinal tract, we are interested in determining whether the hyper-adherent probiotic strain might still displace *Salmonella* even under those conditions. In summary, our findings suggest that this engineered probiotic may function as a non-pathogenic competitive strain capable of displacing or preventing colonization by virulent *Salmonella* serovars through targeted niche occupation and adherence-based exclusion. As pressure increases to reduce antibiotic use in food animal production while simultaneously meeting increasingly stringent food safety standards, this strategy represents an innovative technology for controlling enteric pathogens in poultry. Beyond reducing *Salmonella* carriage in flocks, this work establishes a foundation for the rational engineering of probiotics that are targeted to win colonization competition with pathogens in a given microbial environment to improve animal health, enhance food safety, and reduce the burden of foodborne disease transmission to humans.

## Data Availability

The original contributions presented in this study are included in the article. Further inquiries can be directed to the corresponding author.
